# A new perspective on current prosthetic joint infection classifications: introducing topography as a key factor affecting treatment strategy

**DOI:** 10.1007/s00402-018-3058-y

**Published:** 2018-10-29

**Authors:** Antonio Pellegrini, Claudio Legnani, Enzo Meani

**Affiliations:** 1 IRCCS Istituto Ortopedico Galeazzi, Centre for Reconstructive Surgery and Osteoarticular Infections, Milan, Italy; 2 IRCCS Istituto Ortopedico Galeazzi, Sport Traumatology and Minimally Invasive Surgery Center, Milan, Italy

**Keywords:** Periprosthetic joint infection, Classification, Topography

## Abstract

Periprosthetic joint infection (PJI) is a relatively frequent and devastating complication following prosthetic joint implantation. Several classification systems have been presented by various authors and are routinely used in clinical practice to help in early diagnosis and treatment. The most widely accepted classifications of periprosthetic infections rely on the timing of clinical presentation. Unfortunately, these schemes possess important shortcomings which limit their usefulness in clinical practice, as data reported in literature are contrasting, with success rate ranging from 60 to 80%, irrespectively of prosthetic infection timing. An attempt is made by us to update the current knowledge on PJIs by looking them from a different perspective, introducing a topographic principle in their classification. Our approach is based on the theory that identifying the exact location of the bacterial colonization may allow to decide whether to conservatively treat the patient or to perform a more radical intervention. The aim is to improve the understanding of the aetiology of this serious complication, lead to the appropriate treatment strategy according to the stage of the disease thus enhancing the outcomes of surgical management. Such a strategy, if widely accepted, could guide research studies on the management of PJIs. The availability of investigations like scintigraphy could aid in identifying pathogenetic processes and their exact location, which may be missed on conventional radiographs, and could enable orthopaedic surgeons to have a better understanding of PJI patterns.

## Epidemiology

With growing population and increasing age and activity level, the number of joint arthroplasties performed worldwide is remarkably expanding. Periprosthetic joint infection (PJI) is a relatively frequent and serious complication following prosthetic joint implantation with an average rate of approximately 1–2% [1–6].

## Classification

Several classification systems have been presented by various authors and are routinely used in clinical practice to help in early diagnosis and treatment. All organize such variable as onset of symptoms, causative pathogen and clinical presentation of severity [1, 7–11].

Unfortunately, these schemes possess important shortcomings which limit their usefulness in clinical practice.

All these classification systems are based on the timing of clinical presentation and aim to determine whether or not there is an acute, late, chronic or acute late PJI.

However, classification of acute PJI remains difficult in borderline cases, as the exact definition and cut-off of an acute infection is still unclear ranging between 0 and 4 weeks [8] and 0–3 months [1, 11].

According to current literature, no consensus exists whether a period of 3 months has worse outcome than 4 weeks.

## Treatment

Objectives of treatment are to eradicate the infectious process and to restore the function of the affected limb. According to most widespread guidelines, the treatment of PJI is currently tailored on the degree of disease progression and clinical involvement.

Current treatment options include debridement, antibiotics and implant retention (DAIR), usually recommended at early stages, 1 or 2-stage revision arthroplasty which is commonly advocated at advanced stages.

Since all these approaches use extensive healthcare resources and are extremely expensive, it is mandatory for healthcare practitioner to accurately develop a treatment strategy as much appropriate as possible.

## Limitations of current classification: the sooner, the better?

What remains ambiguous despite existing guidelines is when to perform or not revision surgery.

In fact, the heterogeneity of the results reported in literature poses inherent challenges to clinical decision-making and can lead to uncertainty in whether to perform DAIR or excision arthroplasty.

DAIR is considered to be a valid mean of attempting joint preservation in selected cases, and it is commonly believed that this strategy could achieve satisfying results in patients with a short duration of symptoms [12].

However, its success rate is highly variable among reported studies and it has been estimated that DAIR fails in as much as 44–61% of cases [13–21].

A recent systematic review reported limited success rate for DAIR, even if performed in a selected population of patients, allowing infection eradication in approximately only half of patients at a mean follow-up of 53.3 months [13].

On the other hand, it has been reported that multiple irrigation, debridement, and preservation of components resulted in low morbidity with high success rates in infected joint arthroplasties; Mont et al. reported satisfying outcomes in 10 of the 14 (71%) late hematogenously infected TKAs at an average of 48 months following DAIR [22].

These cases do not reflect any of the available classifications and hence we found the need to look them from a different perspective.

A short duration of symptoms has achieved widespread acceptance internationally as the most predictor of success [13, 17].

However, it has to be kept in mind that the definition of “acute” itself is not clear, partly due to the fact that it is difficult to precisely determine the onset of symptoms, and partly because according to some authors it would occur in less than 4 weeks whereas others suggest less than 6 weeks or even 3 months [1, 8, 11].

Therefore it appears that scientific evidence supporting symptoms duration as the most reliable predictor of outcomes of DAIR is lacking [13–21].

## Introducing topography into current classification systems

Current algorithms do not give univocal advices whenever initial treatment fails and symptoms progressively worsen [1, 2].

With various factors accounting for PJI, it is against clinical logic that timing alone can account as the only factor influencing treatment strategy, and clinical and diagnostic factors other than timing should be recognized as predictors of favourable or unfavourable clinical outcomes.

Recently, some papers have shown acceptable results following revision surgery with partial implant retention in presence of well-fixed prosthetic components in patients with PJI [23–27].

Ekpo et al. [23] reported a re-infection rate of 10% (2 out of 19) in patients undergoing partial revision arthroplasty procedure, at a minimum of 2 years follow-up (range 2–11 years). Similarly, Morley et al. [24] at a mean follow-up of 6.8 years following partial hip revision surgery in 15 patients reported infection recurrence in one patient requiring further revision surgery. El Husseiny and Haddad [26] retained one component in a two-stage revision hip arthroplasty procedure, showing recurrence of infection in three of 18 at a minimum of 5 years follow-up (range 5–9.9 years). Mean Harris hip score was 78 (range 46–89).

**Fig. 1:**
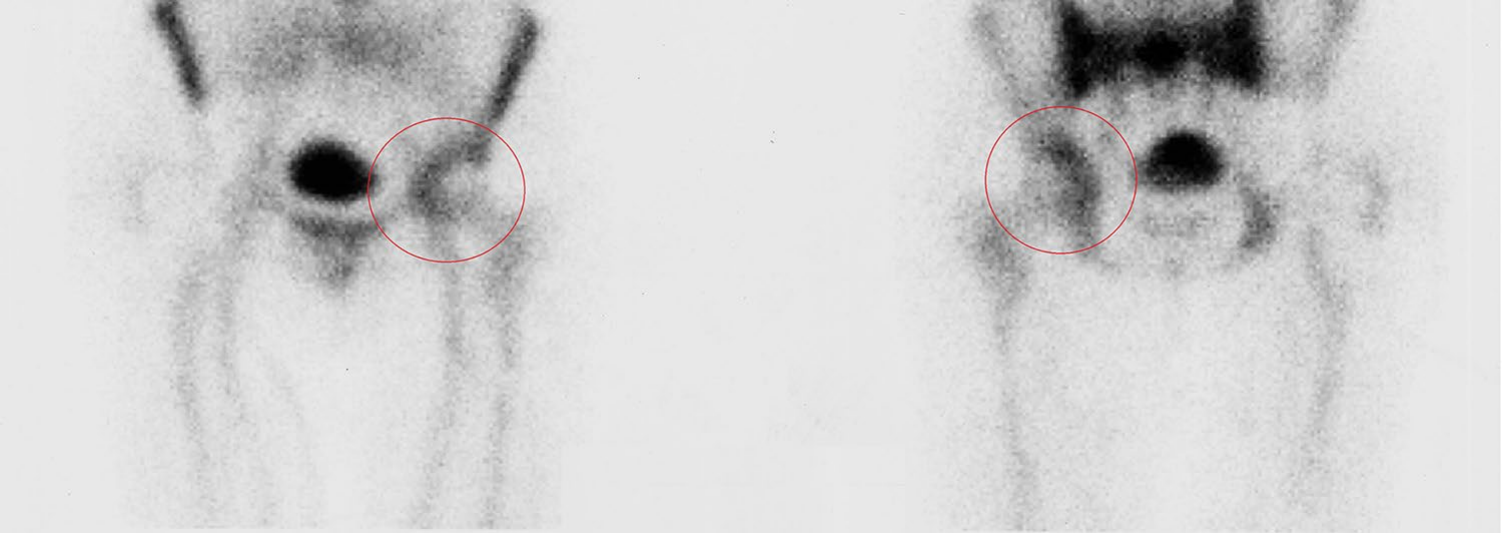
Radiolabeled white blood cells (WBC) imaging showing infection involvement of the soft tissues of the joint space

**Fig. 2:**
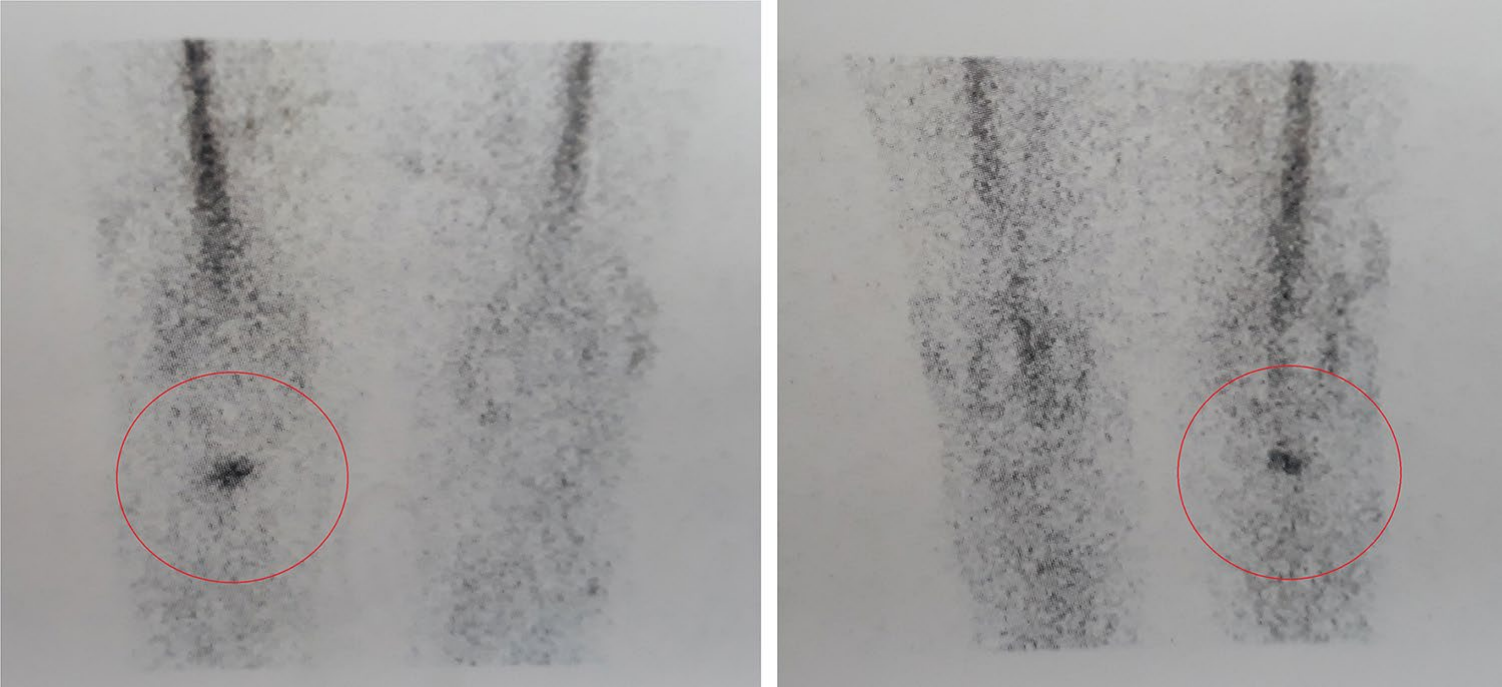
Radiolabeled WBC imaging showing infection involvement of the bone–implant interface

These results may be explained by the fact that not all the prosthetic components are invaded by bacteria, therefore identifying the exact location of the infection may allow the retention of part of the implant without negatively affecting the surgical outcome (Figs. 1, 2).

According to this theory, delayed and chronic infections in which bacterial colonization have not reached the bone–prosthetic interface may not require implant removal, whilst early-diagnosed PJIs in which is demonstrated the presence of infection in the area between bone and implant will probably not benefit from a DAIR procedure, but will require implant removal.

In this way performing early revision surgery would reduce the time and complexity of the surgical intervention, since integration between bone and the implant has not occurred yet.

For these reasons we believe that clinical outcomes could be enhanced by knowing more about the topography of the infectious process.

Our theory concerning topographic staging of the infections suggests that it may allow a better understanding of the infectious process, thus limiting the use of DAIR to those scenarios in which it can be effective for the eradication of the infection, and introducing the need for a more radical approach in those situations in which the establishment of a deep infection of the bone–prosthetic interface requires substitution of the components, even in an early diagnosed infection.

## The role of diagnostic devices

A precocious detection of the infectious process is crucial for a successful treatment [28]. Common symptoms occurring when a PJI is present are usually pain and presence of drainage.

When a PJI is suspected, laboratory tests and joint aspiration are usually performed.

Radiographic assessment may show radiolucent lines, focal osteolysis, or periosteal bone formation. However, these features may not become visible until 2 or 3 weeks after onset of infection. On the other hand, the use of computed tomography (CT) or magnetic resonance imaging (MRI) is limited due to artefacts caused by metallic implants.

Radiolabeled white blood cells (WBC) imaging, with an accuracy of about 90%, its high sensitivity and specificity, is currently the best imaging technique available to discriminate between aseptic loosening and infection [29, 30].

However, recently, in an attempt to improve localization, the role of planar scintigraphy is being overtaken by single-photon emission computed tomography (SPECT) [31].

Compared to planar scintigraphy, SPECT allows more detailed 3D localization, which can provide useful information in patients suspected of having PJI, and recently integrated SPECT/CT has been introduced, allowing high-resolution images with accurate anatomic localization and reported sensitivity, specificity, and accuracy of 93.3% [32].

**Fig. 3:**
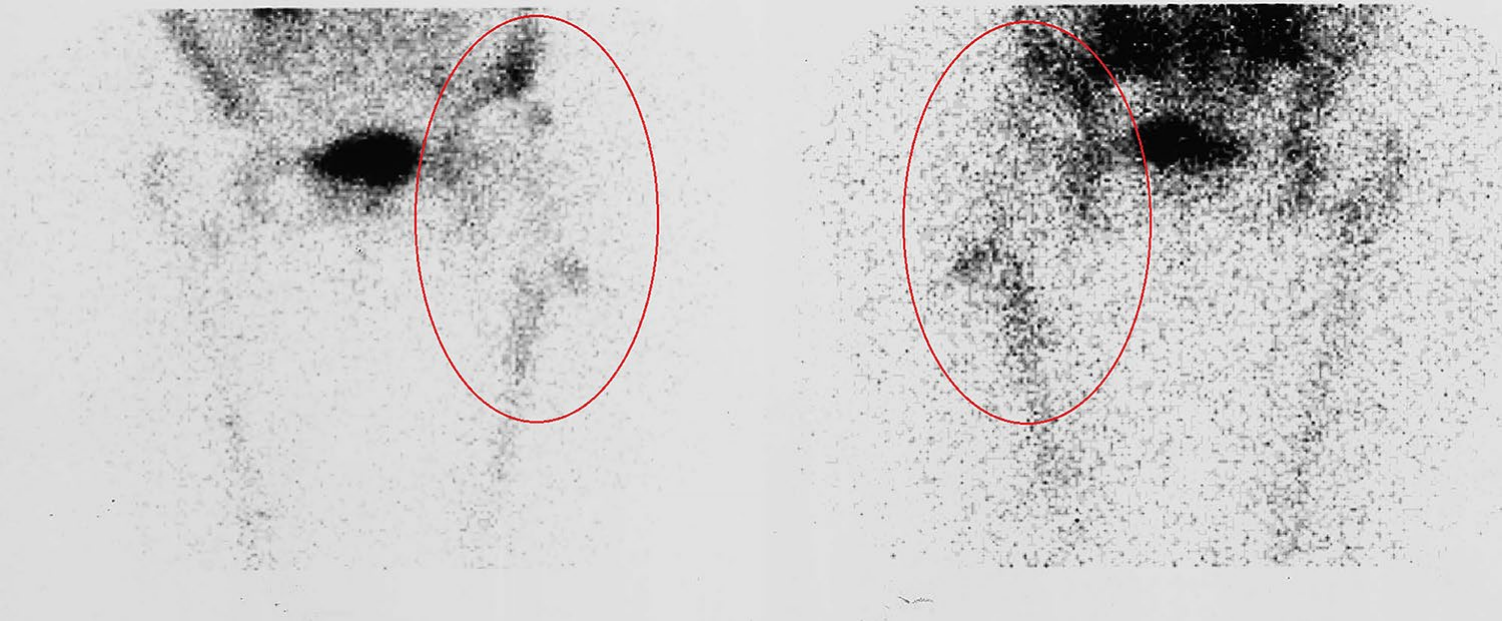
Radiolabeled WBC imaging showing infection involvement of both joint space and bone–implant interface

The use of radiolabeled WBC by SPECT/CT could allow to precisely detect the location of the infection and discriminate between three different patterns:

- infection located in the joint space (Fig. 1)
- infection located at the bone–implant interface (Fig. 2)
- infection involving both compartments (Fig. 3).

According to this new approach, nuclear imaging could support the modification of current patient management concepts and lead to more appropriate selection criteria for surgical treatment, either conservative or radical.

**Fig. 4:**
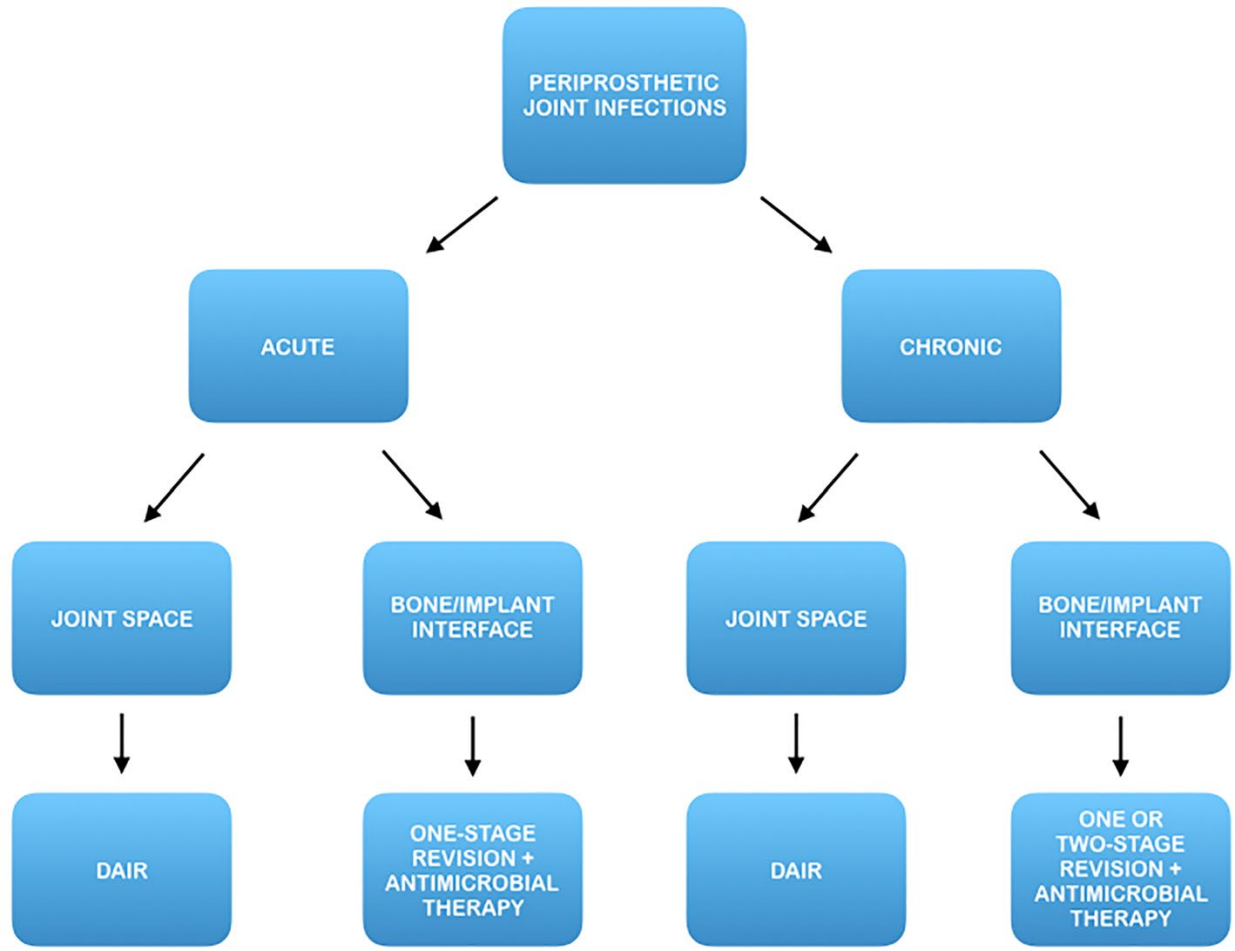
Proposal of an update of current algorithm for prosthetic joint infection treatment introducing topography

In fact, once this topographic distinction has been made, an early prosthetic joint infection located at the bone/implant interface may benefit from a prompt eradicative treatment including implant removal, while a late chronic infection located in the synovial soft tissue could benefit from a DAIR procedure (Fig. 4).

## Future perspectives

Evidence supports that classification schemes are useful for predicting the most appropriate treatment strategy, thus enhancing the outcomes of surgical management.

An attempt is made by us to update the current knowledge on PJIs by looking them from a different perspective, introducing a topographic principle in their classification.

Our approach is based on the theory that identifying the exact location of the bacterial colonization may allow to decide whether to conservatively treat the patient or to perform a more radical intervention. Such a strategy, if widely accepted, could guide research studies on the management of PJIs.

The availability of investigations like scintigraphy could aid in identifying pathogenetic processes and their exact location, which may be missed on conventional radiographs, and could enable orthopaedic surgeons to have a better understanding of PJI patterns.

There are questions which need further evaluation and answering. Whether the newer imaging modalities like nuclear scanning should be included while classifying PJIs is one such question.

This area is worthy of investigation, and the current void of evidence does not allow to confirm or deny the theory that a topographic-centred model should be introduced into current classification systems.

## Conclusions

This paper aims to enrich to the body of current knowledge in the field of PJI by approaching the problem in a unique way, based on the localization of the infective process instead on its timing of appearance.

In addition, it suggests to surgeons and researchers that efforts are needed to improve patients selection by adding a new element into current classification systems.

Treatment standards must no longer rely upon timing only: a philosophy that calls for specific treatments based on the location of the infection must be taken in count.

A strong cooperation between orthopaedic surgeons and radiologist as well as the use of nuclear diagnostic imaging may allow to improve the staging and grading of a PJIs.

With this new perspective in mind, this paper can assist health care professionals who are seeking to identify the most appropriate therapeutic regimen for patients with PJI, enhancing diagnostic tools and optimizing clinical outcomes.
